# A consortia of clinical *E. coli* strains with distinct in vitro adherent/invasive properties establish their own co-colonization niche and shape the intestinal microbiota in inflammation-susceptible mice

**DOI:** 10.1186/s40168-023-01710-y

**Published:** 2023-12-20

**Authors:** Rachel M. Bleich, Chuang Li, Shan Sun, Ju-Hyun Ahn, Belgin Dogan, Cassandra J. Barlogio, Christopher A. Broberg, Adrienne R. Franks, Emily Bulik-Sullivan, Ian M. Carroll, Kenneth W. Simpson, Anthony A. Fodor, Janelle C. Arthur

**Affiliations:** 1 Department of Microbiology and Immunology, University of North Carolina at Chapel Hill, Chapel Hill, NC USA; 2 Present Address: Department of Biology, Appalachian State University, Boone, NC USA; 3 College of Computing and Informatics, University of North Carolina at Charlotte, Charlotte, NC USA; 4 Department of Clinical Sciences, Cornell University College of Veterinary Medicine, Ithaca, NY USA; 5 Center for Gastrointestinal Biology & Disease, University of North Carolina at Chapel Hill, Chapel Hill, NC USA; 6 Lineberger Comprehensive Cancer Center, University of North Carolina at Chapel Hill, Chapel Hill, NC USA

**Keywords:** Inflammatory bowel disease, Colitis, AIEC, Adherent-invasive *E. coli*, Mucosal colonization, Intestinal microbiota, Interleukin-10-deficient mouse

## Abstract

**Background:**

Inflammatory bowel disease (IBD) patients experience recurrent episodes of intestinal inflammation and often follow an unpredictable disease course. Mucosal colonization with adherent-invasive *Escherichia coli* (AIEC) are believed to perpetuate intestinal inflammation. However, it remains unclear if the 24-year-old AIEC in vitro definition fully predicts mucosal colonization in vivo. To fill this gap, we have developed a novel molecular barcoding approach to distinguish strain variants in the gut and have integrated this approach to explore mucosal colonization of distinct patient-derived *E. coli* isolates in gnotobiotic mouse models of colitis.

**Results:**

Germ-free inflammation-susceptible interleukin-10-deficient (*Il10*^*−/−*^) and inflammation-resistant WT mice were colonized with a consortium of AIEC and non-AIEC strains, then given a murine fecal transplant to provide niche competition. *E. coli* strains isolated from human intestinal tissue were each marked with a unique molecular barcode that permits identification and quantification by barcode-targeted sequencing. 16S rRNA sequencing was used to evaluate the microbiome response to *E. coli* colonization. Our data reveal that specific AIEC and non-AIEC strains reproducibly colonize the intestinal mucosa of WT and *Il10*^*−/−*^ mice. These *E. coli* expand in *Il10*^*−/−*^ mice during inflammation and induce compositional dysbiosis to the microbiome in an inflammation-dependent manner. In turn, specific microbes co-evolve in inflamed mice, potentially diversifying *E. coli* colonization patterns. We observed no selectivity in *E. coli* colonization patterns in the fecal contents, indicating minimal selective pressure in this niche from host-microbe and interbacterial interactions. Because select AIEC and non-AIEC strains colonize the mucosa, this suggests the in vitro AIEC definition may not fully predict in vivo colonization potential. Further comparison of seven *E. coli* genomes pinpointed unique genomic features contained only in highly colonizing strains (two AIEC and two non-AIEC). Those colonization-associated features may convey metabolic advantages (e.g., iron acquisition and carbohydrate consumption) to promote efficient mucosal colonization.

**Conclusions:**

Our findings establish the in vivo mucosal colonizer, not necessarily AIEC, as a principal dysbiosis driver through crosstalk with host and associated microbes. Furthermore, we highlight the utility of high-throughput screens to decode the in vivo colonization dynamics of patient-derived bacteria in murine models.

Video Abstract

**Supplementary Information:**

The online version contains supplementary material available at 10.1186/s40168-023-01710-y.

## Introduction

Inflammatory bowel disease (IBD) affects over one million people in the USA and costs billions of dollars in direct medical expenses annually [1]. IBD-linked chronic intestinal inflammation defines both Crohn’s disease (CD) and ulcerative colitis (UC), which currently have no medical cure. Additionally, IBD patients with longstanding inflammation experience a greater risk of developing inflammation-associated colorectal cancer (CRC) [2–4]. The resident microbes in the intestine are implicated in both IBD and CRC in humans and mouse models. This dysbiotic state of the gut microbial community includes an increased abundance of *Escherichia coli* (*E. coli*) and reduced microbial diversity [5–11].

Intestinal tissues of IBD and CRC patients often harbor high loads of mucosal adherent/invasive *E. coli* (AIEC), which are believed to perpetuate intestinal inflammation [10, 12–14]. AIEC are distinguished by functional attributes tested through exhaustive in vitro co-culture assays, based on the ability to adhere to and invade epithelial cells and survive in macrophages [9, 12, 15], the latter of which has recently been shown to correlate strongly with in vivo inflammation [16]. However, it has been difficult to assess AIEC abundance across patient populations, because AIEC do not contain a universal genetic signature and cannot be screened molecularly from other gut-resident *E. coli* [17–19]. This limitation has prevented us from a comprehensive understanding of how the AIEC pathobiont takes control of microbiome community composition and interactions with the host, and what molecular features permit colonization of the inflamed mucosa. This information is important for us to identify IBD patients colonized with high-risk *E. coli* strains, but might be hindered by inaccurate in vitro classification. Instead, reclassification based on in vivo phenotype (i.e., mucosal colonization) can potentially bridge this gap.

The mammalian intestine contains at least two distinct bacterial communities: the luminal and mucosally adherent [20]. These two microbial communities differ in composition and function, and it is the mucosal community that readily interacts with intestinal epithelial and mucosal immune cells. It is well-documented in IBD patients and animal models that disturbance or dysbiosis of the mucosal community strongly impacts health and disease. In fact, a microbiome study on 447 CD patients and 221 unaffected controls revealed disease-associated dysbiosis of the mucosally adherent, but not the fecal, microbiota in CD patients [21]. Notably, increased mucosally adherent bacteria and bacterial translocation are observed in IBD patients and mouse models [10, 12–14, 22]. Several lines of evidence underscore the importance of mucosal interactions in microbial-driven disease. Both *pks* + *E. coli* and *cagA* + *Helicobacter pylori* must have physical contact with mammalian cells to exert their pro-carcinogenic activities [23, 24]. Mucosal biofilms promote colorectal cancer in mouse models [25], and in humans are populated by potentially pro-carcinogenic bacteria including *pks* + *E. coli*, enterotoxigenic *Bacteroides fragilis* and *Fusobacterium spp.* [4]. We have recently described that siderophore production by pathobiont *E. coli* NC101 promotes tissue colonization and inflammation-associated fibrosis [26, 27]. As the mucosal niche positions bacteria in an ideal location to exert pro-inflammatory and pro-carcinogenic activities, it is essential for us to predict which resident microbes are likely to colonize the mucosa of IBD patients.

The gut *E. coli* characteristics of AIEC (defined in vitro) and mucosal colonization (observed in vivo) are two determinant factors in IBD. Although both phenotypic traits are prevalent in IBD, how AIEC/non-AIEC pathotypes vary in their ability to colonize mucosa remains to be fully understood. To clarify this, we sought to determine the extent to which the in vitro AIEC definition predicts mucosal colonization in vivo, and whether that specific colonizing consortia can contribute to a dysbiotic event in gut microbiota. We hypothesize that mucosally adherent *E. coli* consortia, defined as either AIEC and/or non-AIEC strains, utilize common functional capabilities and genomic features to colonize and play a central role in modulating the crosstalk between inflamed host and mucosal microbiome during the progression to a dysbiotic state.

Here we describe a polymicrobial colonization strategy that uses a novel barcoding technology to easily distinguish genetically similar, but functionally distinct, *E. coli* strains in a complex community*.* We have incorporated this strategy with a collection of well-characterized clinical AIEC and non-AIEC strains isolated from human intestinal tissues and a well-established IBD mouse model to profile microbiome community dynamics. Our findings demonstrate that non-AIEC strains can colonize together with AIEC, but with variable patterns in different host contexts. Guided by mucosal colonization status, molecular features unique to in vivo colonizers were identified and regarded as genomic advantages for colonization. We separated the luminal from the mucosal microbiota to highlight *E. coli* and microbial assembly that occurs in these distinct niches within the colon. High levels of mucosally co-colonized non-AIEC and AIEC strains promoted structural alterations and loss of diversity in the mucosal microbiome within the inflamed intestines, which is normally associated with a dysbiotic state. Collectively, diversified *E. coli* colonization patterns mark the evolutionary response of mucosal colonizers to host selection and microbiome interactions by utilizing their conserved metabolic advantages.

## Methods

### Bacterial strains

We utilized seven clinical *E. coli* strains that were isolated from ileal tissue of Crohn’s disease and non-Crohn’s disease patients [11, 14, 28]. Strains were classified as AIEC or non-AIEC using standard in vitro assays to evaluate adhesion/invasion to Caco2 colonic epithelial cells and uptake/survival in J774 macrophages [9, 14]. These strains were referenced by their blinded laboratory designation: JA0018, JA0019, JA0022, JA0036, JA0044, JA0048, and JA0091 [28]. The identity of these strains is in Table 1. Five strains were originally isolated by KW Simpson at Cornell University as the strains CU39ES-1, CU532-9, CU568-3, CU37RT-2, and CU42ET-1. HM670 was gifted by Barry Campbell from Liverpool University [10], and LF82 was gifted to KW Simpson by Arlette Darfeuille-Michaud [9].

**Table 1:** Characteristics of seven *E. coli* isolates used in study. Note: N indicates the strain defined as non-AIEC; Y indicates the strain defined as AIEC

Strain code	Strain names	Phylogency	AIEC	*ybt*	Disease origin	Barcode
JA0018	CU39ES-1	B2	N	*ybt*^+^	Ileal CD	A1
JA0019	CU532-9	B2	N	*ybt*^+^	Ileal CD	A3
JA0022	LF82	B2	Y	*ybt*^+^	Ileal CD	B6
JA0036	CU568-3	B2	Y	*ybt*^+^	Colonic CD	C5
JA0044	CU37RT-2	B2	N	*ybt*^+^	non-IBD	D2
JA0048	CU42ET-1	B2	Y	*ybt*^+^	non-IBD	D5
JA0091	HM670	B2	N	*ybt*^+^	Colon cancer	C2

### Bacterial growth conditions

Bacteria were grown in Luria broth (LB) at 37 °C and 250 rpm unless otherwise indicated. Barcoded strains were selectively plated on LB with kanamycin. Serial dilution plating to enumerate fecal and tissue-associated CFU were performed as described [27].

### Insertion of molecular barcode

To track individual strains of *E. coli* from among our complex microbiota, each strain was marked with a kanamycin antibiotic resistance cassette and molecular barcode inserted into a neutral chromosomal region using site-specific Tn7 transposon insertion [28–30]. Barcode designations are listed in Table 1 with a schematic in Fig. S1.

### Validation of AIEC phenotype in barcoded strains

#### Cell culture

Human colonic epithelial cell line Caco-2 (ATCC HTB-37) were grown in minimum essential medium (Gibco, Rockville, MD, USA) supplemented with 15% fetal bovine serum (FBS), 1 mM sodium pyruvate, and 0.1 mM nonessential amino acid solution (NEAA). Murine macrophage-like cell line J774A.1 (ATCC TIP-67) were cultured in Dulbecco’s modified Eagle’s medium (DMEM, Gibco) supplemented with 10% FBS. Cells were cultured at 37 °C in a humidified incubator with 5% CO_2_ in air.

#### Invasion of cultured intestinal epithelial cells

The invasive abilities of *E. coli* isolates were evaluated in cultured Caco-2 epithelial cells by the gentamicin protection assay. Caco-2 cells were seeded at 2 × 10^5^ in 24-well plates, grown for 2 days and infected with overnight cultures of *E. coli* strains at a multiplicity of infection (MOI) of 10 for 3 h. Intracellular bacteria were determined as described previously [31]. A non-invasive *E. coli* strain (DH5α) and an invasive *E. coli* strain (541–15) were used as negative and positive controls, respectively.

#### Persistence and replication within macrophages

To assess the survival and replication of *E. coli* isolates within macrophages, the standard uptake and survival assay was conducted, as previously described [14, 31] with some modifications. Briefly, J774 cells were seeded at 2 × 10^5^ in 24-well plates and infected at a MOI of 10, and the plates were centrifuged at 1200 rpm 7 min. After 1 h incubation, cells were washed three times with PBS, and fresh medium containing 50 μg/ml of gentamicin was added to kill extracellular bacteria. Following an additional 1 h of incubation, cells were washed once with PBS and lysed in 1 ml of 1% Triton X-100 (Sigma-Aldrich) in deionized water for 5 min to assess uptake. In parallel, to measure intracellular survival beyond 24 h post-infection, fresh cell culture medium containing 15 μg/ml gentamicin was added. Bacteria were diluted and plated onto LB agar plates to determine the number of CFUs. A non-invasive *E. coli* strain (T75) and an invasive *E. coli* strain (541–1) were used as negative and positive controls, respectively. Survival was expressed as the mean percentage of bacteria present within cells at 24 h compared to the number uptake at 1 h (100%). Each experiment was performed in triplicate and repeated at least two times.

### Animal care

Germ-free mice were reared in the National Gnotobiotic Rodent Resource Center at UNC Chapel Hill. Once colonized, mice were maintained in sterile cages in an isolation cubicle under specific pathogen-free (SPF) housing conditions. All animal experiments and procedures were approved by UNC’s Institutional Animal Care and Use Committee (IACUC).

### Murine FMT preparation

Fecal microbial transplant 1 (FMT1) was prepared anaerobically from 7 C57BL/6 WT specific pathogen-free (SPF) mice that were *Helicobacter spp.* free. Briefly, colonic and cecal content were removed in an anaerobic chamber and resuspended in sterile, reduced PBS to make a slurry. The slurry was homogenized by vortexing and physical disruption with a filter pipette. Glycerol was added to 15% final concentration before aliquoting and storing at − 80 °C. Fecal microbial transplant 2 (FMT2) was prepared in the same manner from 7 germ-free C57BL/6 WT male mice that were colonized via oral gavage with 100 µl of FMT1. This serial FMT preparation strategy was applied to enhance reproducibility in notoriously noisy microbiome datasets, and quite simply because we could not easily find a second colony of C57BL/6 mice that were Helicobacter-negative in the same mouse facility as our isolation (not gnotobiotic) cubicles, we had no detected endogenous *E. coli* in the FMT1 inoculum, and we did not want to introduce a new microbiome from another facility or supplier.

### Murine model

**Fig. 1:**
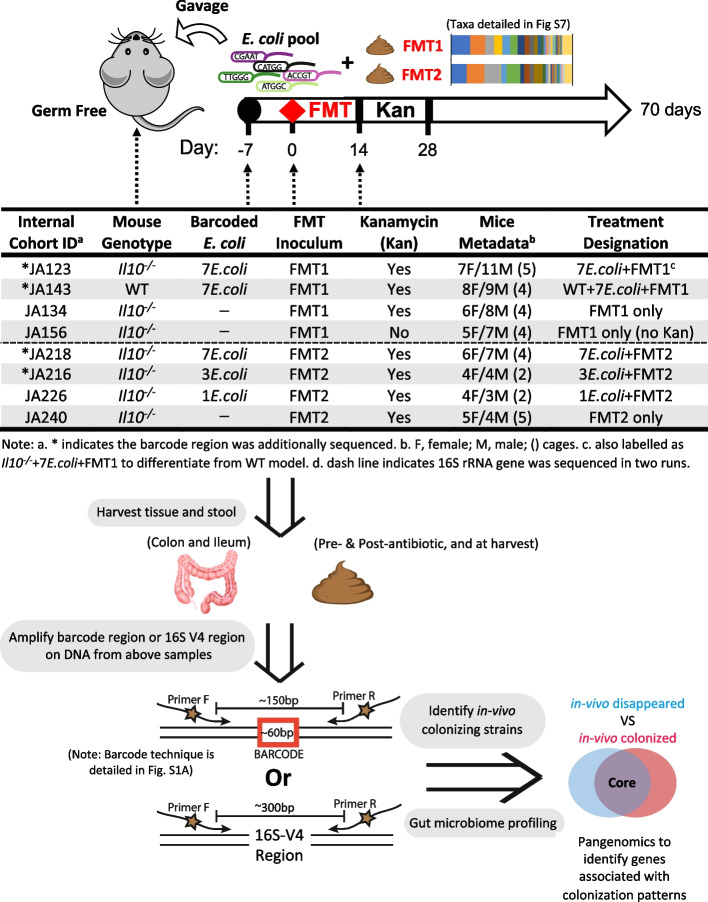
Workflow for novel high-throughput in vivo approach to identify highly colonizing *E. coli* strains and structural shifts in the mucosally adherent intestinal microbiome. Germ-free mice (inflammation-susceptible *Il10*^*−*/*−*^ and inflammation-resistant WT) were gavaged with various pools of barcoded clinical *E. coli* strains followed by fecal microbial transplants (FMT) from pooled WT C57Bl/6 mice. We reference each experimental group by their cohort ID and treatment designation (i.e., WT + 7*E.coli* + FMT1). Tissue and stool were harvested from each cohort. DNA was extracted from harvested samples and used as template to PCR amplify the barcode region identifying each *E. coli* strain and V4 region of 16S rRNA gene for microbiome analysis. Illumina sequencing of barcoded regions revealed highly colonizing *E. coli* strains at the mucosally adherent niche for further pangenomic analysis to pinpoint candidate colonization factors

**Fig. 2:**
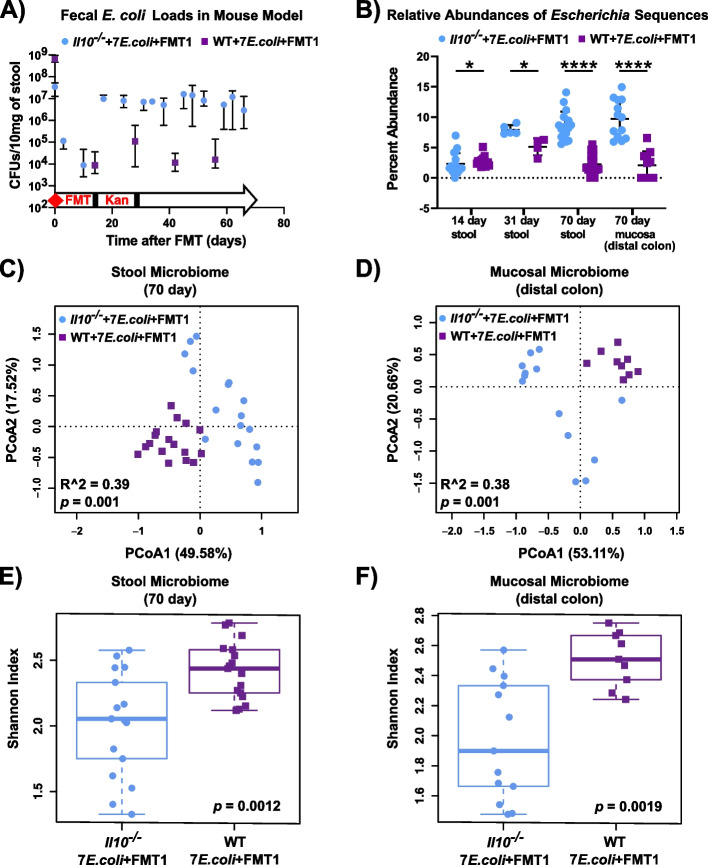
Inflamed (*Il10*^*−/−*^) vs. un-inflamed (WT) states influence *E. coli* mucosal colonization and the associated stool and mucosal microbiomes. *Il10*^*−/−*^ (blue) and WT (purple) mice were maintained under the same condition (7 *E. coli* isolates and FMT1, see Fig. 1). **A** CFUs of *E. coli* in stool over time, assessed by serial dilution plating. Dots and error bars respectively indicate the median with a 95% CI. **B** Abundances of *Escherichia* 16S sequences relative to total microbiota 16S sequences in stool at three time points and in distal colon mucosa at harvest. Data was normalized and log-transformed. Each symbol represents an individual mouse (**B**–**F**). Line is at mean, and *p*-values determined by Mann–Whitney test (* *p* < 0.05, **** *p* < 0.001). **C**, **D** PCoA based on Bray–Curtis distance for the stool and mucosal microbiota in WT and *Il10*^*−/−*^ mice harvested at sacrifice (*p* = 0.001, *n* = 9–17 mice per group). **E**,** F** Microbiome diversity as measured by Shannon index (*p* < 0.01, *n* = 9–17 mice per group) for the stool and mucosal microbiota in WT and *Il10*^*−/−*^ mice. Box and whisker plots show the median, first/third quartiles, and min/max index values

In these studies, we used the established interleukin-10 deficient (*Il10*^*−/−*^) mouse model [3, 32], described in detail in Fig. 1. *Il10*^*−/−*^ (inflammation-susceptible) and wild-type (inflammation resistant) 129S6/SvEv mice were reared germ-free to adulthood (8–10 weeks) in the cohorts listed in Fig. 1. Mice were colonized with an even mixture of a total of 10^8^ CFU of the barcoded *E. coli* strains and maintained in an isolation cubicle in SPF housing. One week after colonization, mice were given 100 µl of thawed FMT for colonization competition with a normal, murine microbiota. Two weeks post-FMT, we gave kanamycin water (0.4 g/L) ad libitum for 2 weeks to suppress the microbiota and ensure that some of the barcoded *E. coli* strains could persist to the end of our model. We had noted in pilot studies that *E. coli* quickly became undetectable in some WT mice without the addition of kanamycin (Fig. 2A). In addition, germ-free mice housed in the same isolation cubicle on kanamycin water did not become colonized with any kanamycin-resistant aerobes able to grow on LB (i.e., *E. coli* and similar *Enterobacteriaceae*). Throughout the experiment, *E. coli* loads per gram of stool were determined by serial dilution and quantitative culture on LB-kanamycin selection plates. Mice were harvested by CO_2_ asphyxiation after a total of 10 weeks colonization post-FMT. Stool samples were removed from the lumen and after flushing contents with sterile PBS, 1 cm of colonic tissue (mucosal sample) was taken for sequencing analysis. Mice that received just FMT were treated the same as above without the 1 week of colonization with barcoded *E. coli* strains. Some *Il10*^*−/−*^ mice in internal cohort designation JA123 that received barcoded *E. coli* with FMT1 required premature sacrifice due to concerns for their care as they appeared hunched and in poor health, as can occur in this colitis model. Early sacrifices happened: mouse 17—day 5 post FMT due to rectal prolapse, mice 12–14—day 36 post FMT, mice 5–11—day 55 post FMT. Histological analysis revealed no abnormalities in the colon. Pro-inflammatory cytokine expression analysis of proximal colon tissue revealed higher *Il6* expression in mice sacrificed early, while comparable expression of most other cytokines permitted us to include them together with mice sacrificed normally in the downstream analysis (Fig. S2). We repeated this experiment with the internal cohort designation JA218 (7 *E. coli* + FMT2) and no mice required early sacrifice, while another two cohorts JA216 (3 *E. coli* + FMT2) and JA226 (1 *E. coli* + FMT2) were colonized with 3 (CU39ES-1_A1, CU568-3_C5, CU42ET-1_D5) and 1 (CU42ET-1_D5) strains that were selected from the *E. coli* pool. Male and female mice were both used. As in our previous studies with *Il10*^*−/−*^ mice, we observed no sex effect on colitis severity or microbiome. Some mice from certain cohort were not included for specific downstream analysis due to inevitable loss from abnormal animal death, PCR failure, and/or low sequencing depth.

### Colon histology

At necropsy, the majority of the colon not stored for sequencing analysis was swiss rolled with distal colon in the center of the roll, fixed in 10% neutral buffered formalin, sectioned and stained with H&E. Colitis scores (0–4) of rolled colons were blindly assessed by an experienced investigator, as previously described [3, 27, 32].

### DNA extraction, libraries preparation (16S rRNA & E. coli Barcode) and Illumina sequencing

DNA was isolated from murine fecal and mucosal samples using a phenol/chloroform extraction with physical disruption, followed by clean-up using the Qiagen DNeasy Blood and Tissue extraction kit as previously described [3, 28, 33, 34]. Amplification of the variable V4 region of the 16S rRNA gene (515–806 bp) via a two-step PCR strategy to create libraries for Illumina MiSeq PE-250 sequencing was performed as previously described [35]. Amplification of the molecular barcode occurred via the same two-step PCR strategy, but using primers targeting the conserved area flanking the unique barcode region in the first round of amplification (F-5′-ATCTCCACTAGTTACCTACAACCTCAAGCT-3′, R-5′-CTGCAAACTAGTTACCCATTCTAACCAAGC-3′), with a primer-binding (P2/P4) schematic in Fig. S1. Both libraries were sequenced at the UNC High Throughput Sequencing Facility. Raw sequencing reads were processed and analyzed as described in a subsequent section.

### Sequencing analysis and statistical analysis

The 16S rRNA sequencing generated 52 million reads and *E. coli* barcode sequencing generated 20 million reads. The DADA2 and Quantitative Insights Into Microbial Ecology 2 (QIIME2) pipeline were used for processing 16S rRNA sequencing reads. The forward and reverse reads were denoised using DADA2 “denoise-paired” method, and the chimera were removed using DADA2 “consensus” method. The samples with low sequencing depth (≤ 2000 reads) were discarded. Taxonomy was assigned using QIIME2 feature classifier “classify-sklearn” based on SILVA databases (release 132). Molecular barcodes were assigned using BLAST with a threshold of 99% similarity. The taxonomic abundance tables were normalized to correct for different sequencing depth across samples using a previously reported method [36]. Bray–Curtis dissimilarity between samples were calculated using normalized abundance of genera and visualized using principal coordinate analysis (PCoA). The differences in microbial composition between groups were analyzed with PERMANOVA test using R function “adonis” with 999 permutations. Alpha diversity was calculated using Shannon diversity index and the differences in Shannon diversity were analyzed with Wilcoxon test, and visualized with box and whisker plot. Correlations between *Escherichia* and other genera were calculated with Spearman’s correlation. The calculation was based on all the stool and mucosa samples in each of five specified cohorts, and the abundance was normalized to the same depth across samples to limit the effect of sequencing bias. *P*-values were adjusted with the Benjamini–Hochberg method to correct for multiple hypotheses testing. Biological data (histology scores and cytokine expression) were assessed for significance between two groups using the nonparametric Mann–Whitney test. *P*-values of < 0.05 were considered significant.

### Pangenomic analyses

To characterize the distribution of the gene content, comparative analysis of the genomes of seven *E. coli* isolates was performed through the pangenomics pipeline in Anvi’o v7.1 [37]. The similar pipeline was also applied to characterize the genotypic diversity of gut *Akkermansia* [38]. Here, the sequenced genomes of *E. coli* isolates were described in our previous study [28] and retrieved from NCBI under the BioProject accession number PRJNA759208. An Anvi’o-compatible contig database was constructed from each of genome fasta files using the command anvi-gen-contigs-database. This command uses Prodigal [39] for gene prediction and then KEGG KOfam database [40] for function assignment with the script parameter anvi-run-kegg-kofams. To facilitate the downstream comparison on gene sequence, the annotated contig databases were subsequently combined and converted to a single genome database by running anvi-gen-genomes-storage. To compare the genes for presence/absence among genomes (known as “pangenomes”), the command anvi-pan-genome was run based on BLASTP comparisons between all pairs of encoded amino acid sequences from all seven *E. coli* strains, followed by construction of clusters of homologous genes (designated as “gene cluster”) with an MCL inflation factor of 10 for very closely related genomes. The created pangenome was visualized in an interactive interface by running the script anvi-display-pan, where seven isolates are clustered based on gene frequencies and the phylogram is clustered based on presence/absence pattern of gene clusters. Based on the observed colonization patterns of *E coli* strains in GI tissue in the context of mice model: *Il10*^*−/−*^ + 7*E.coli* + FMT1, each isolate was assigned to the colonized or disappeared group (see details in result) using the script anvi-import-misc-data. Group-specific KEGG gene functions, represented by the ones present in subset or all members of a given group and absent in the other group, were obtained using the command anvi-compute-functional-enrichment-in-pan. Initial pangenome comparisons were made between two colonization groups on the basis of KEGG profiled functions. The KEGG orthology database could fail in homology assignments for certain gene families due to the lack of archived orthologous templates, while further searching against another database is possible to achieve successful function annotation. Thus, the gene cluster with unknown functions in KEGG were extracted using anvi-split and checked for annotation against NCBI Clusters of Orthologous Groups (COG) [41]. The COG-annotated gene clusters were then used as input to identify the group-specific COGs. To better display the numbers and shared patterns of group-specific gene functions (annotated by KEGG or COG), UpSet plots were created using the UpSetR package in R [42]. More broadly, comparisons at a high level for group-specific functions, represented by BRITE hierarchies [43] in KEGG and COG categories, were summarized in the supplemental material.

## Result

### Novel barcoding approach allows tracking in vivo colonization of clinical* E. coli *strains in a murine model

Cost-effective techniques that enable us to profile bacterial consortia at the strain level in host-associated tissues are still lacking. To discriminate closely related bacterial strains from mucosa in a high-throughput manner, we developed an approach that uses a Tn7 transposon system to molecularly tag strains through site-specifically inserting a unique genomic barcode and kanamycin resistance cassette into a neutral chromosomal region **(**Fig. S1A). The incorporated tag facilitates quick identification/enumeration with PCR/qPCR using barcode-specific primers, or quantification with high-throughput sequencing using primers for conserved regions outside of the barcode (Fig. S1A). We confirmed that barcode insertion did not impact AIEC status for each strain listed in Table 1 using validated assays to define AIEC phenotype: epithelial invasion and survival in macrophages (Fig. S1B, C) [12, 14, 31].

Murine *E. coli* NC101 is a well-studied model AIEC strain with pro-inflammatory and pro-carcinogenic activities [3, 32, 44]. To validate Illumina sequencing can be applied to read and quantify the barcodes, NC101 was tagged with 10 different barcodes to obtain 10 uniquely barcoded clones. We pooled clones with various known proportions in vitro, which were subsequently processed in the same manner as traditional 16S sequencing but treating the barcode as a “variable” region. Percent ratio of 10 barcode reads calculated from sequencing were nearly identical with the corresponding expected ratios in each of five different mixtures (Fig. S3A). This outcome demonstrates that we have no technique barrier for barcode sequencing in vitro mock communities. To further test if the barcoding approach impacts initial strain colonization, three germ-free mice were gavaged with evenly pooled five barcoded NC101 clones and their stools were harvested after 8 h (*n* = 1) or 24 h (*n* = 2) based on the estimated in vivo transit time (Fig. S3B). Barcode-targeted qPCR of three stool samples showed even colonization levels of five barcoded clones in stools (Fig. S3C); that observation was confirmed by Pielou’s evenness index (Fig. S3D). Consistent evenness, as harbored in inoculum, supports that there is no bottleneck for strains to achieve successful colonization at the initial stage. Once we extended the sampling time to 1 week after inoculating with the same five NC101 clones, sequencing the stool samples from 13 mice showed minor shift on an expected even colonization ratio, likely due to in vivo selective pressure over time (Fig. S3E).

To evaluate the effectiveness of this approach in a physiologically relevant system, we utilized seven clinical *E. coli* strains (Table 1) in an equal proportion to colonize 17 gnotobiotic mice. A murine fecal transplant was provided after 1 week to provide niche competition (depicted as JA123 in Fig. 1). The clinical *E. coli* strains were from a well-characterized culture collection isolated from intestinal mucosa of IBD and non-IBD patients [11]. The particular seven strains were chosen from among the B2 phylogroup that includes *ybt* + and *pks* + strains, which we have previously shown can impact IBD-associated fibrosis and CRC [3, 27, 45]. Stools were collected after 10 weeks to characterize the colonization capabilities of *E. coli* strains by measuring their presence and relative abundance. Barcode-targeted PCR revealed that three strains of *E. coli* with barcode A1/C5/D5 can colonize consistently across most of mice (Fig. S4A), in line with their high abundance revealed through qPCR of the molecular barcode (Fig. S4B). The sequencing obtained a similar result, with the same three strains as the dominant colonizers at the lumen niche (Fig. S4C). We verified that barcode insertion did not affect the growth rate by comparing to each *E. coli* parent strain, ensuring the insertion event had no impact on bacterial fitness and proliferation (Fig. S4D). Overall, our high-throughput barcode sequencing permits the accurate assignment of the sequence origin from among related intestinal *E. coli* strains and paves the way to evaluate their in vivo colonization dynamics at the mucosal niche in a murine model.

### Host genotype (inflamed vs. un-inflamed) impacts colonization of clinical *E. coli* consortia and the intestinal microbiomes

To evaluate colonization dynamics of clinical *E. coli* strains under distinct inflammation states, we developed an in vivo model using inflammation-susceptible *Il10*^*−/−*^ and inflammation-resistant WT germ-free mice gavaged with seven barcoded *E. coli* isolates premixed at equal ratio. An identical fecal transplant (FMT1) was given to all recipient mice after 1 week to initiate niche competition. Mice were subsequently maintained on kanamycin water for 2 weeks to ensure measurable amounts of *E. coli* persisted in WT mice, which historically harbor low *E. coli* loads (JA123 and JA143 in Fig. 1). Fecal *E. coli* levels of both cohorts were monitored throughout the 10-week experimental period by serial plating on LB-kanamycin plates to select for transplanted strains. As expected, *E. coli* bloomed over time to significantly higher levels in *Il10*^*−/−*^ mice, which was approximately 2-log greater than in WT mice by the experiment’s end (Fig. 2A). Meanwhile, the V4 region of the 16S rRNA gene was amplified from fecal DNA samples at three time points and from the colon mucosa at harvest. Calculation of *Escherichia*-belonging sequences revealed similar colonization dynamics as serial plating, showing a significant increase of *Escherichia* load in stool and mucosa of *Il10*^*−/−*^ mice as compared to WT mice (Fig. 2B). This elevated *E. coli* load in an inflammation-susceptible host is consistent with observations on the gut microbiome of mouse models and CD patients [21].

We next sought to characterize the structure of colonic luminal and mucosal microbiomes of *Il10*^*−/−*^ and WT mice after 10 weeks of colonization. Bray–Curtis distances visualized in PCoA plot showed that fecal and mucosal communities in *Il10*^*−/−*^ mice significantly differed from those in WT mice (Fig. 2C,D). As noted in the methods, some *Il10*^*−/−*^ mice in this cohort required early sacrifice as they exhibited poor health from colitis; we provide duplicate PCoA plots and average colitis histology score indicating harvest time post FMT (Fig. S2). *Il10*^*−/−*^ mice also harbored a significantly reduced microbial diversity compared to WT mice (Fig. 2E,F). These results thus far support previous observations that host genotype influences *E. coli* abundance, and also the structure and diversity of the fecal and mucosal microbiomes.

**Fig. 3:**
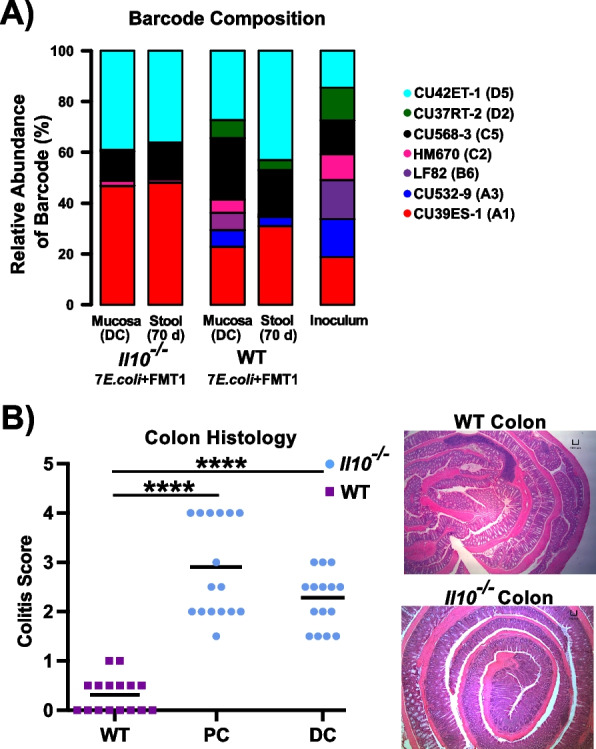
Inflamed (*Il10*^*−/−*^) vs. un-inflamed (WT) states promote divergent patterns of *E. coli* colonization in the stool and mucosa. *Il10*^*−/−*^ and WT mice were the same as Fig. 2. **A** Column graph showing relative abundance of 7 *E. coli* strains in inoculum, distal colon mucosa and stool collected at the harvest, based on analyzing the barcode region. **B** Inflammation of the proximal colon (PC) and distal colon (DC) in *Il10*^*−/−*^ mice was scored histologically (0–4) as compared to WT mice (Each symbol in WT represents PC and DC score averaged together). Line is at mean, and *p*-values determined by Mann–Whitney test compared to WT (**** *p* < 0.001). Representative H&E histology at × 40 of the colon. Scale bar = 100 µm

We next determined the composition of *E. coli* consortia in the same DNA samples from above two cohorts using barcode-targeted sequencing. After inoculation with the equally pooled clinical *E. coli* consortia, *Il10*^*−/−*^ mice exhibited a loss of diversity relative to WT mice **(**Fig. 3A), indicating host genotype might also affect colonization patterns of *E. coli*. We validated that in response to *E. coli* and FMT colonization, the two cohorts *(Il10*^*−/−*^ and WT) exhibited distinct inflammation states. Histopathology revealed significant levels of intestinal inflammation in *Il10*^*−/−*^ mice but not in WT mice, as expected in this IBD model (Fig. 3B). Since we administered kanamycin from weeks 1 to 3 to improve the engraftment of the barcoded clinical strains, we evaluated the impact on the microbiome after 10 weeks. We compared two cohorts of *Il10*^*−/−*^ mice that were both gavaged the FMT1, with one cohort administered kanamycin and one not (JA134 and JA156 in Fig. 1). Shannon diversity of the stool and mucosal microbiome was not significantly different between the two cohorts (Fig. S5A, B), and colonic inflammation remained the same (Fig. S5C), indicating kanamycin’s impact on the microbiome and inflammation is negligible. In summary, the inflammatory micro-environment that develops in *Il10*^*−/−*^ mice promotes selective expansion of *E. coli* strains with high colonization potential and re-structuring of the intestinal microbiota.

### High colonization with *E. coli* significantly alters the microbiome in the inflamed intestine

**Fig. 4:**
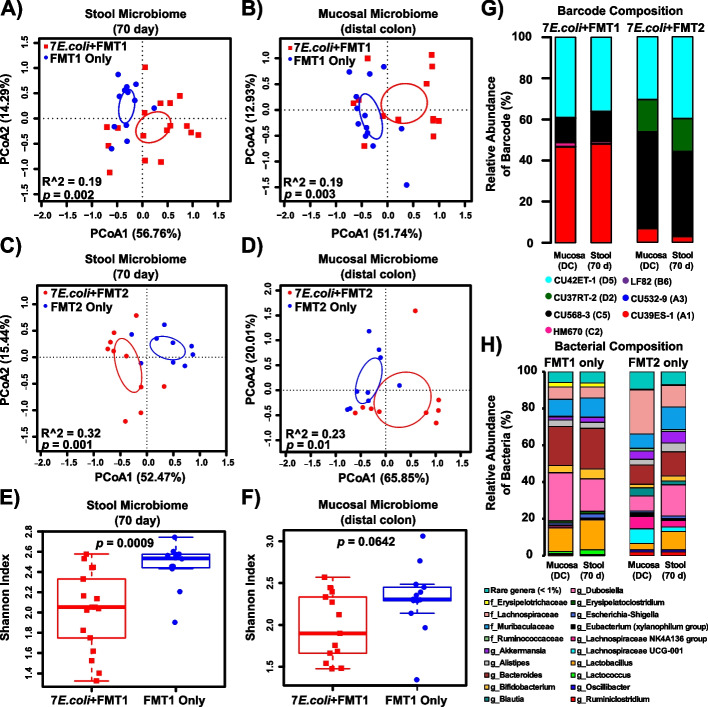
Colonization by the *E. coli* consortia alters the structure of the stool and mucosal microbiome of the inflamed colon.** A**, **B** PCoA based on Bray–Curtis distance for the stool (**A**) and mucosal (**B**) microbiome in *Il10*^*−/−*^ mice that received FMT1 with (red) or without (blue) *E. coli*. **C**, **D** PCoA based on Bray–Curtis distance for the stool (**C**) and mucosal (**D**) microbiome in *Il10*^*−/−*^ mice that received FMT2 with (red) or without (blue) *E. coli*. **E**, **F** The Shannon diversity index for the stool (**E**) and mucosal (**F**) microbiome in *Il10*^*−/−*^ mice that received FMT1 with (red) or without (blue) *E. coli*. Each symbol represents an individual mouse, *n* = 9–17 mice per group. Circles in **A**–**D** represent 95% confidence limits. Box and whisker plots show the median, first/third quartiles, and min/max index values. **G** Relative abundance (% of total barcoded reads) of 7 barcoded *E. coli* strains colonizing the distal colon mucosa and stool of *Il10*^*−/−*^ mice that received FMT1 or FMT2. Bars associated with *Il10*^*−/−*^ cohort “7*E.coli* + FMT1” are also included in Fig. 3A for another comparison. **H** Microbiome composition of the distal colon mucosa and stool in *Il10*^*−/−*^ mice that received FMT1 or FMT2 without *E. coli*. Rare genera with < 1% abundance in any of the samples were collapsed together. Unassigned sequences that could not be classified with accuracy at the phylum level were removed; g—genus, f—family

The inflamed *Il10*^*−/−*^ mice colonized by high loads of *E. coli* experienced structural alterations in the intestinal microbiota, leading us to examine whether the bacterial community directly responded to the *E. coli* expansion. We utilized the *Il10*^*−/−*^ cohort described above that received a pool of barcoded *E. coli* consortia, FMT1, and kanamycin, and a parallel cohort treated identically but without inoculation of *E. coli* (JA123 and JA134 in Fig. 1). PCoA analysis based on 16S rRNA gene sequences revealed overlapping but significantly different clustering of both the stool and mucosal microbiomes between mice that received *E. coli* and FMT1 and those only received FMT1 (Fig. 4A,B). To confirm these results, we repeated the experiment with another two cohorts that were treated identically, except given a different fecal microbial transplant (FMT2) (JA218 and JA240 in Fig. 1). Stool and tissue samples were collected and sequenced in the same manner in an independent sequencing run. Consistent with results from FMT1, *Il10*^*−/−*^ mice colonized with *E. coli* and FMT2 versus FMT2 alone had significantly different stool and mucosal microbiomes (Fig. 4C,D). Correspondingly, significantly decreased microbial diversity was observed in mice that had added *E. coli* (Fig. 4E,F). Measurements of colonic tissue cytokine expression demonstrated that *Il10*^*−/−*^ mice colonized with either FMT1 or FMT1 + 7 *E. coli* exhibited high inflammatory cytokine expression (Fig. S6). We also note that both cohorts of *Il10*^*−/−*^ mice colonized with either FMT1 + 7 *E. coli* or FMT2 + 7 *E. coli* developed high histologic inflammation scores at 2.38 ± 0.10 and 2.62 ± 0.19 respectively (displayed as mean ± SEM), characteristic of this colitis model. Together, our results demonstrate that high loads of *E. coli* contribute to microbial community changes in the inflamed intestine.

Beyond microbiome profiling, we continued to determine the colonization patterns of the clinical *E. coli* consortia in the above two cohorts of *Il10*^*−/−*^ mice (JA123 and JA218 in Fig. 1). Barcode sequences were amplified from the same stool and mucosal DNA templates to quantify each individual strain. Four *E. coli* strains became the obviously outcompeting colonizers in each cohort after 10 weeks of colonization. Specifically, three strains (CU42ET-1_D5, CU568-3_C5, and CU39ES-1_A1) were maintained in both *Il10*^*−/−*^ cohorts with a different proportion, while HM670_C2 and CU37RT-2_D2 were unique to either of one cohort (Fig. 4G). To better understand how this differential composition developed from evenly pooled *E. coli* consortia, we checked temporal dynamics of their surrounding microbes (represented by FMT1 or FMT2 in our mice model). The microbial compositions were moderately comparable in the initial inoculum of FMT1 vs. FMT2, based on similar taxonomic proportions and a potential positive correlation on lineage abundances (Fig. S7A, B). However, the two FMTs appear to have evolved differently over time in vivo, as they progressed into distinct constitutions at the final colonization stage in mice (JA134 and JA240 in Fig. 1; Fig. 4H). These differences were probably driven by stochastic events such as housing/air or cage effect [46]. Overall, our results suggest that the initial FMT communities experienced divergent ecological succession in *Il10*^*−/−*^ mice, which likely influenced the colonization dynamics and outcomes of the co-evolved *E. coli* consortia.

**Fig. 5:**
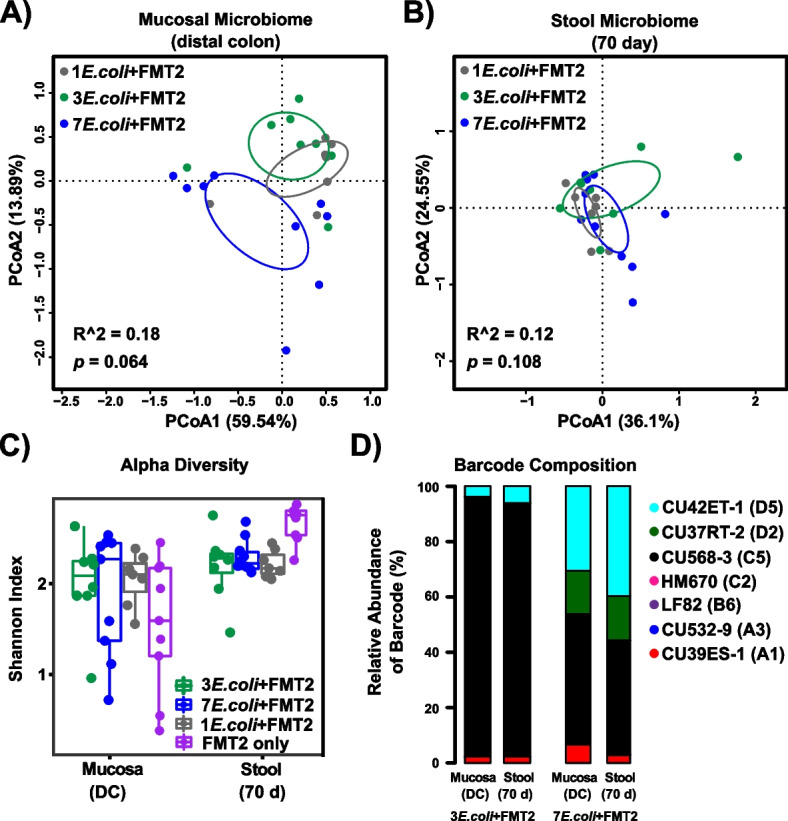
The mucosal and stool microbiota respond similarly to inoculation with different numbers (1, 3, or 7) of *E. coli* strains, whereas the abundance of unique *E. coli* strains differs by the number of originally inoculated strains.** A**–**C** PCoA based on Bray–Curtis distance for the mucosal (**A**) and stool (**B**) microbiome and Shannon diversity (**C**) in *Il10*^*−/−*^ mice that received 7, 3, or 1 *E. coli* isolate(s) followed by FMT2. **A**, **B** Each symbol represents an individual mouse, and circles represent 95% confidence limits. Box and whisker plots show the median, first/third quartiles, and min/max index values. There were no significant differences between three cohorts with *E. coli*-colonization. **D** Column graphs show relative abundance of barcoded *E. coli* strains colonizing the distal colon mucosa and stool in *Il10*^*−/−*^ mice that received 3 or 7 *E. coli* isolates followed by FMT2. Bars under cohort “7*E.coli* + FMT2” are also included in Fig. 4G for another comparison

We questioned whether a high abundance of *E. coli* comprised of 7 strains would elicit similar effects on the microbiome as colonization with fewer distinct *E. coli* strains. To test this, we repeated our *E. coli* + FMT colonization of *Il10*^*−/−*^ mice using either 1, 3, or 7 *E. coli* strains followed by FMT2 (JA218, JA216 and JA226 in Fig. 1). The structure of both the stool and mucosal microbial communities were minimally affected by the initial diversity (1, 3, or 7 *E. coli* strains) of the colonizing *E. coli* consortia (Fig. 5A,B). This observation was also supported by pairwise comparisons (Fig. S8). Furthermore, *E. coli* strain diversity in the initial inoculum did not significantly impact microbiome diversity (Fig. 5C). As for the related *E. coli* colonization pattern, the predominantly colonizing isolate CU568-3_C5 dramatically decreased in mice as the numbers of inoculated strains increased (Fig. 5D), implying intense niche competition occurs among this *E. coli* consortia over 10 weeks of colonization in the inflamed intestines.

Altogether, these results indicate that high colonization with *E. coli*, regardless of the numbers of distinct strains, exerts strong selective pressure in shaping the structure and biodiversity of the stool and mucosal microbiome in the inflammation-susceptible host. Additionally, microbial interactions among *E. coli* consortia and/or with other members of the microbiota shape the intestinal *E. coli* colonization patterns.

### Intestinal bacteria associated with* E. coli *strains under specific in vivo context

**Fig. 6:**
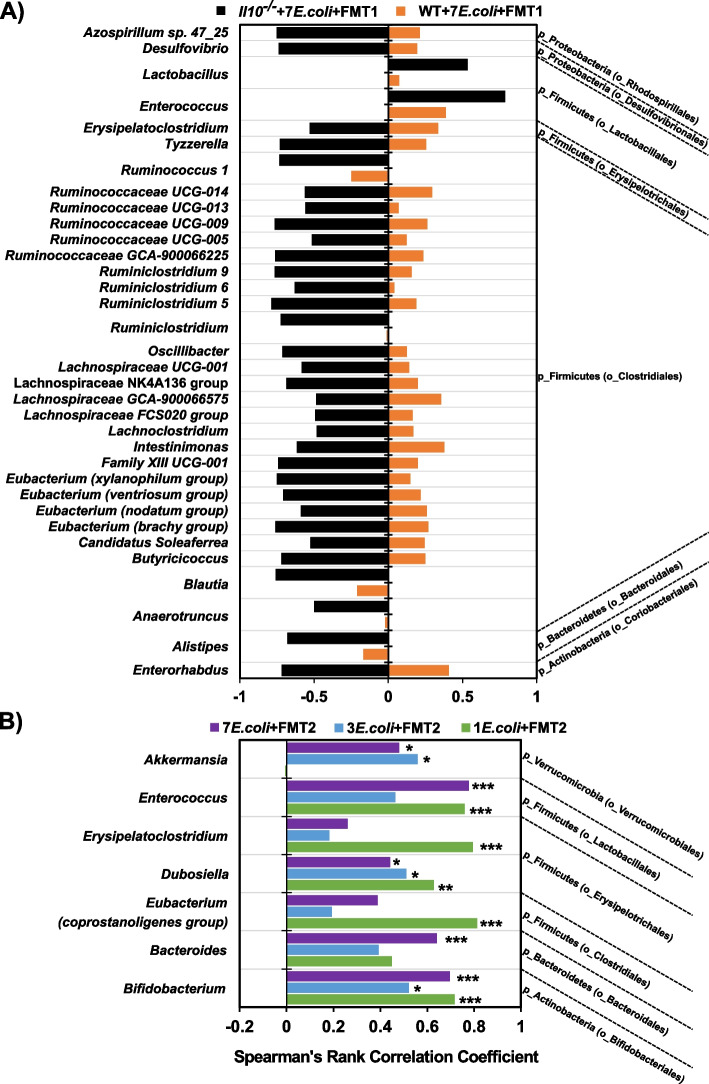
Co-occurring genera with *Escherichia* in (**A**) WT vs *Il10*^*−/−*^ mice colonized with 7 *E. coli* isolates and FMT1 (**B**) *Il10*^*−/−*^ mice colonized with 7 vs 3 vs 1 *E. coli* isolate(s) and FMT2. The coefficient rho values from 0 to ± 1 in the bottom axis represent the degree of the correlation from weak to strong. Minus value (on left) indicates a negative correlation while plus value (on right) represents a positive correlation. **A** Only the genera with correlation significances of FDR-corrected* p* < 0.001 in *Il10*^*−/−*^ + 7*E.coli* + FMT1 (black bars) are displayed, while none of these genera significantly correlated in WT + 7*E.coli* + FMT1 (orange bars). **B** Only the genera with correlation significances of FDR-corrected *p*-values less than 0.05 in any of the 3 cohorts are listed (*** *p* < 0.001; ** *p* < 0.01; * *p* < 0.05). The genera are further collapsed into p—phyla and o—order levels on the right of the graph

Given that *E. coli* community assembly was compositionally diverse under the various in vivo conditions described above (Figs. 3A, 4G, and 5D), we next aimed to identify which taxa bloomed and contracted in response, as these associated members are likely candidates that influence *E. coli* colonization patterns. In inflamed *Il10*^*−/−*^ mice, *E. coli* abundance was negatively correlated with the abundance of 32 genus-level bacterial lineages and positively correlated with another 2 lineages. However, no genera correlated with *E. coli* abundance in uninflamed WT mice (Fig. 6A), suggesting inflammation is required for these dynamics. Furthermore, such contrary correlation patterns are consistent with the microbiome shift observed between host genotypes and in response to inflammation-induced *E. coli* expansion (Fig. 2A–D). These correlations also reveal potential interacting partners that drive the observed *E. coli* colonization pattern in the inflamed intestine (Fig. 3A). To determine if these positive and negative correlations between the *E. coli* consortia and inflammation-associated microbiome differed by the diversity of the initially colonizing *E. coli* consortia, we compared *Il10*^*−/−*^ mice colonized with either 1, 3, or 7 *E. coli* strains followed by FMT2. Here we observed a similar positive correlation between the abundance of *E. coli* and 7 other taxa among all three cohorts (Fig. 6B). In concordance with PCoA results (Fig. 5A,B), this suggests that the intestinal microbiome responds to mucosally colonizing *E. coli* regardless of their strain diversity. Furthermore, with the similarity in microbiomes, these data strongly support that niche competition within *E. coli* consortia is a strong driver of strain-specific colonization of the mucosa, as displayed in Fig. 5D. Another important finding in these data is that, except for sharing a significantly positive correlation of *E. coli* abundance with *Enterococcus* abundance, no correlating taxa were shared between *Il10*^*−/−*^ mice that received FMT1 or FMT2 (*Il10*^*−/−*^ + 7*E.coli* + FMT1 vs. 7*E.coli* + FMT2 in Fig. 6A,B and Fig. S9). This observation supports our earlier interpretation that the FMT1 and FMT2 communities experienced distinct evolutionary paths that can impact colonization dynamics of the associated *E. coli* consortia (Fig. S7A-B; Fig. 4G,H). Collectively, we conclude that *E. coli* strain colonization patterns (Figs. 3A, 4G, and 5D) are impacted through microbe:*E. coli* and *E. coli*:*E. coli* interactions encountered in specific in vivo contexts.

### The mucosal colonization patterns of AIEC and non-AIEC strains

The strains of our *E. coli* consortia include three AIEC and four non-AIEC (Table 1). Given the association between AIEC and IBD, most notably in CD, we determined whether strains in vitro defined as AIEC had a higher colonization capacity in the inflamed *Il10*^*−/−*^ intestines. *Il10*^*−/−*^ mice inoculated with FMT1 retained four strains at the mucosa, including two AIEC (CU568-3_C5 & CU42ET-1_D5) and two non-AIEC (CU39ES-1_A1 & HM670_C2), thus no AIEC preference existed in this cohort over 10 weeks of colonization (Fig. 3A). In the FMT2-colonized *Il10*^*−/−*^ cohort, two AIEC (CU568-3_C5 & CU42ET-1_D5) and two non-AIEC (CU39ES-1_A1 & CU37RT-2_D2) outcompeted three other strains to almost undetectable levels (Fig. 4G). Non-AIEC strains are able to colonize the *Il10*^*−/−*^ mice mucosa: non-AIEC strain CU39ES-1_A1 colonized to high levels in the presence of competing community FMT1, and non-AIEC strain CU37RT-2_D2 colonized to high levels in the presence of competing community FMT2 (Fig. 4G). Thus, in vitro defined AIEC status cannot explain these mucosal colonization patterns. The uninflamed environment of WT mice appeared more hospitable to *E. coli* colonization, as all seven strains persisted in the mucosal microbiome to some extent (Fig. 3A). Importantly, while the diversity of *E. coli* strains was higher in the uninflamed WT mucosa, *E. coli* were significantly less abundant (2-log fold) than in the inflamed *Il10*^*−/−*^ intestines. Together these data suggest that colonization dynamics of individual AIEC and non-AIEC strains are more likely modulated by inflammation states and the composition of the competing microbiome, rather than in vitro-defined AIEC status.

### Common genomic features of clinical *E. coli* strains that colonize the inflamed mucosa

*E. coli* colonization is strongly influenced by intrinsic metabolic potential that may be conserved across highly colonizing strains. To identify potential genomic features that drive colonization of the inflamed intestines, we grouped clinical *E. coli* strains under two terms: “colonized” and “disappeared.” Three *E. coli* strains (CU39ES-1_A1, CU568-3_C5 and CU42ET-1_D5) had colonized to high levels. Despite lower abundance, strain HM670_C2 widely persisted in colonic tissues and was also included in the “colonized” group (Fig. 3A and S10A-B). Thus CU39ES-1_A1, CU568-3_C5, CU42ET-1_D5, and HM670_C2 comprise the “colonized” group, while the remaining strains were assigned the “disappeared” group. The CD-associated AIEC strain LF82 was among the “disappeared” group and served as an internal control. Although LF82 can induce inflammation in murine models [47, 48], LF82 poorly adheres to the mouse epithelium, attributed to lack of murine CEACAM6 expression [48, 49]. Mannosylated CEACAM6 is the binding partner for FimH, an *E. coli* type I pili-associated adhesin with high polymorphism, and thus variable binding to mammalian cell membrane-associated ligands, among AIEC strains [50–52]. Therefore, we would expect luminal (stool) but not mucosal colonization of LF82, as observed (Figs. 4G and 5D).

**Fig. 7:**
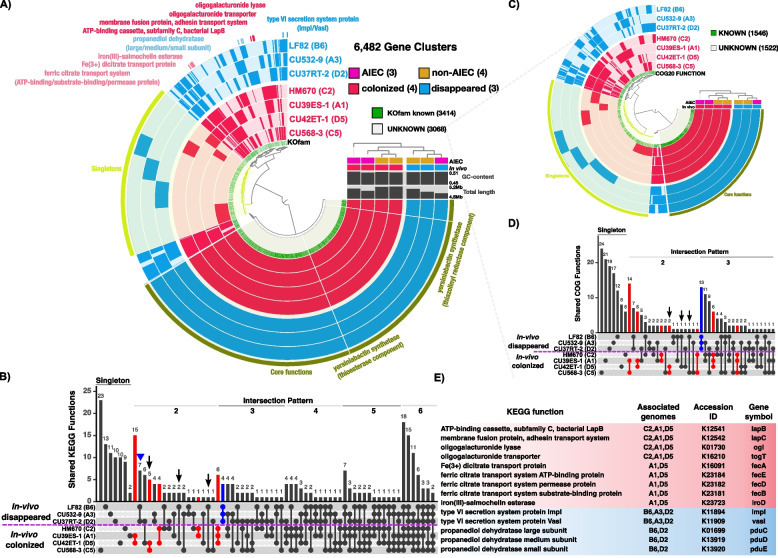
Pangenomic analysis reveals gene features associated with *E. coli* in vivo mucosal colonization of the inflamed intestine.** A** Circle phylogram displays the pangenome of four in vivo “colonized” isolates (red) and three in vivo “disappeared” isolates (blue), showing gene cluster presence/absence (solid/light color in the layer) by isolate. In vitro defined AIEC and non-AIEC are denoted with pink and orange squares. Gene clusters with assigned KEGG functions are labelled with green, or gray for unknown. Yersiniabactin synthetase gene groups contained in core genomes are common to all the isolates and serve as a positive control. Selected group-specific gene clusters are highlighted to show that their distribution was confined to either “colonized” (red) or “disappeared” (blue) groups. **B** UpSet plot displays KEGG gene functions associated with *E. coli* strains in “colonized” or “disappeared” groups. Columns of the matrix show intersection patterns of KEGG functions: a single dot denotes non-shared functions within that isolate (singleton pattern), with linked dots showing numbers of functions shared and exclusive to those linked isolates (shared pattern 2 to 6 displayed; core pattern 7 not shown). The vertical bar shows frequency of functions exclusive to those isolate(s). The blue bar depicts number of functions present in all disappeared isolates and absent in colonized isolates, while red denotes number of functions detected in 2 to 3 colonized isolates and absent in all disappeared isolates. Arrows reveal the number of functions exclusively shared by AIEC strains. Three gene functions unique to two disappeared strains (see triangle-pointing pattern) are discussed in the main text. **C** Circle phylogram displays the pangenome of 7 *E. coli* isolates based on further exploration of KEGG unannotated gene clusters against COG database. **D** UpSet plot displays COG gene functions associated with specific “colonized” (red) or “disappeared” (blue) groups. The arrow reveals the number of COG functions exclusively shared by AIEC strains. **E** The list of selected KEGG functions displayed in phylogram and their associated isolate genomes displayed in UpSet plot. Red: functions specific for “colonized” isolates. Blue: functions specific for “disappeared” isolates. The full list of group-specific gene functions is contained in Table S1

To identify genes that may promote colonization of the inflamed intestines, we performed a pangenome analysis on the seven *E. coli* isolates. We identified a total of 6482 gene clusters, with 3585 core gene clusters shared among all genomes and 1469 gene clusters found only in single genomes (Fig. 7A). These results indicate that *E. coli* can adapt to diverse host niches with a large and flexible gene pool. We were particularly interested in specific accessory genes present in most or all members of the “colonized” group that were absent in the “disappeared” counterparts. These genes represent candidate colonization factors to be further explored in future studies. To identify them, we compared the distribution of KEGG-annotated gene functions between two defined groups. This approach highlighted 31 gene functions specific to “colonized” and 4 gene functions specific to “disappeared” strains (red and blue bars in Fig. 7B). We next queried common gene functions among in vitro defined AIEC, regardless of their colonization capacity, and identified 8 gene functions (arrows in Fig. 7B). We identified only 8 unique gene functions shared among in vitro-defined “AIEC,” underpinning the lack of genomic biomarkers in AIEC. To overcome the annotation limit of single database, the 3068 gene clusters with unknown KEGG functions were further explored with COG database to facilitate comprehensive comparisons. Accordingly, we analyzed the pangenome of the same strains but narrowed it down to this undefined gene pool, from which 1546 gene clusters were successfully assigned with COG functional categories (Fig. 7C). Consistently, “colonizers” contained much higher numbers of group-specific COG functions than AIEC strains (red bars vs. arrows in Fig. 7D).

The group-specific gene functions annotated by either KEGG or COG databases represent potential colonization factors (Fig. 7E and Table S1). Three “colonized” strains (HM670_C2, CU39ES-1_A1, CU42ET-1_D5) putatively encode a distinct subclass of ATP-binding cassette (ABC) transporters that were absent in “disappeared population.” The transporter components, including a cytoplasmic membrane-localized ATPase LapB and a membrane fusion protein LapC, are known to participate in the secretion of large adhesin to cell surface to facilitate biofilm development [53]. Another unique gene product in this “colonized” group, DNA helicase IV (HelD) can lead to the formation of a biofilm with filamentous morphology in *E. coli* [54]. The same three strains appear capable of utilizing energy-rich pectin from diet, due to possession of a series of pectinolytic enzymes (PelB and PelX) that sequentially cleave polygalacturonate to form oligogalacturonides [55]. Oligogalacturonides can be transported in through the TogT transporter [56] and metabolized by oligogalacturonides lyase Ogl [57], all present among three “colonized” genomes. Enzymes necessary for catabolic conversions of sucrose (ScrY/A/R and SacA) were identified as an exclusive mark of “colonized” CU42ET-1_D5 and CU568-3_C5 [58]. “Colonized” strains CU42ET-1_D5 and HM670_C2 harbored the *pgtABCP* operon encoding a phosphoglycerate transporter system that mediates the transport of glycolysis intermediates across the cytoplasmic membrane [59, 60]. These versatile mechanisms for carbohydrate metabolism may provide members of the “colonized” consortia access to a broad range of small carbon molecules for growth and persistence in the intestine.

Iron acquisition genes permitting additional strategies for iron acquisition were a feature of the dominant “colonizers” CU42ET-1_D5 and CU39ES-1_A1. Both genomes harbored a ferric citrate ABC transport system consisting of *fecABDELR* genes that assists to uptake ferric dicitrate complex through the cytoplasmic membrane [61–63]. They also harbored genes for salmochelin conversion, a glycosylation of the ubiquitous siderophore enterobactin that is catalyzed by glucosyltransferase IroB. This modification allows bacteria to circumvent capture by the host immunity protein lipocalin-2 [64] and, while characteristic of *Salmonella* species, has also been found in some intestinal *E. coli* strains [64–66]. Cytoplasmic and periplasmic esterases, encoded by iroD and iroE, allow the following cleavage of iron-bound salmochelins, that is required for iron release and salmochelins reutilization [66]. This suggests that they might use multiple ways to compete with host and microbes for scavenging iron and promote growth during infection.

Unexpectedly, a key feature of the “disappeared” group is the Type VI secretion system (T6SS) that is dedicated to the delivery of toxins into neighboring bacteria and host cell [67]. We identified two T6SS proteins, ImpL and VasI, assigned through KEGG and then supplemented these findings with eight COG-predicted T6SS components, demonstrating the advantage of using independent annotation systems to assign previously unknown KEGG functions. Another unexpected contributor to differences in colonization phenotypes may be the catalytic activity of propanediol dehydratase PduC/D/E to generate propionate [68], which is present only in two “disappeared” strains (indicated by triangle in Fig. 7B). The presence of such well-conserved genomic features among colonization groups may represent inherent molecular mechanisms permitting or inhibiting colonization of the mucosa. However, future in vivo colonization studies employing many more *E. coli* strains coupled with direct investigation of each molecular mechanism (i.e., knockout strains) will be necessary to truly define conserved colonization factors.

## Discussion

### A high-throughput in vivo system to track colonization dynamics of strain-level variants

*E. coli* represent a diverse group of bacteria with pathogenic, commensal, and probiotic activities. Techniques to distinguishing such closely related but functionally divergent strains from within a complex host-associated environment are still lacking. The common strategy of sequencing variable regions of the 16S gene is insufficient to resolve genetically similar strains [69, 70], while metagenomics with higher resolution largely fails at the tissue niche from contaminating host material [71]. A compromised method sequencing on the entire rRNA operon achieves desirable classification accuracy, whereas much higher error rates of long-read sequencing should not make this as a general metataxonomic analysis procedure [28]. Due to technique limitations, our field has been unable to efficiently and effectively link bacterial identity to their in vivo functional and pathogenic potentials at the strain level. We have developed a high-throughput sequencing approach to quantify individual *E. coli* strains by incorporating a unique barcode sequence into a neutral genomic region, and demonstrated this molecular barcode permits accurate identification and discrimination of each clone from in vitro mock mixtures and in vivo IBD mouse models. Similar methods have been used to track the translocation of pathogenic bacteria (uropathogenic *E. coli* and *Yersinia pestis*) during infection in mouse models; however, barcode detection and quantification was limited by inefficient techniques like qPCR or Southern dot blot [29, 30]. Our results demonstrate that barcode-targeted high-throughput sequencing can effectively resolve seven clinical *E. coli* strains, with results in concordance with barcode-targeted PCR and qPCR. This approach, which can be applied to consortia of tens or hundreds of strains, will permit a rapid, reliable, and thorough means to assess the colonization capacity of individual *E. coli* strains in order to better define high-risk IBD-associated *E. coli.* In combination with in vivo fluorescent imaging, our method would facilitate connecting spatial dynamics to functional activities from both qualitative and quantitative perspectives [29, 72]. One obvious limitation of our genetic engineering approach is common in microbiology: the candidate strains must be culturable with known genomes in order to properly engineer the molecular barcode or fluorescence markers.

### Human intestinal tissue-derived* E. coli *can colonize and induce inflammation in the *Il10*^*−/−*^ mouse model

For human-derived *E. coli* strains to stably colonize mice, they must be capable of adapting to different epithelial surface structures in mice. Previous studies have revealed certain human fecal-derived lineages have a poor transfer rate into GF mice [73]. Therefore, we tested the colonization capacity of seven *E. coli* strains isolated from human intestinal biopsies—thus all strains had the ability to colonize human intestinal mucosa. All strains were retained in the luminal compartment (feces) of at least some animals after 10 weeks (Fig. S4A), demonstrating stable colonization. We observed distinct colonization patterns for mucosally adherent *E. coli* communities across disease states (uninflamed WT and inflammation-susceptible *Il10*^*−/−*^ mice) and in the presence of different competing microbial communities (FMT1 and FMT2). This illustrates human-derived *E. coli* strains can colonize GF mice, although to varying extents based upon both inherent ability and microenvironment. Inoculating human-derived strains into murine model in the setting of a complex microbial community, rather than in mono-association with murine *E. coli* strain or fecal slurry used in previous studies [3, 32, 45, 74], elevates the physiological relevance of our model. Accordingly, the expansion of these *E. coli* colonizing strains is linked to a significant inflammatory response and microbiome shift in *Il10*^*−/−*^ mice, in line with previous results [3, 21, 75–78]. These coincident consequences demonstrate that colonized human *E. coli* strains can retain the same functional characteristic and utility in murine intestinal environment.

### Crosstalk between the host, *E. coli* and microbiome drives intestinal dysbiosis in the inflamed gut

Many reports have described the co-occurrence of intestinal inflammation, *E. coli* expansion, and reduced microbiome diversity in the intestine of *Il10*^*−/−*^ mouse model [77], yet it has been difficult to break apart their cause-and-effect relationships. Our data and the previous research support an ongoing dialog between host and microbes, where inflammation fuels expansion of *E. coli* and microbiome changes, which in turn exacerbate inflammation [74, 77]. Importantly, we illustrate here that this communication is occurring at the mucosal barrier, a key site of host:microbe interactions [79]. However, it is still unclear whether microbiome changes are directly driven by host inflammation or inflammation-induced *E. coli* expansion. We have thus compared microbiomes of two cohorts colonized with or without intestinal *E. coli*. PCoA analyses illustrated that high colonizing loads of *E. coli*, but not numbers of distinct introduced strains, shapes the structure of microbiome in an inflamed environment, in concert with the observation that many microbes co-shifted with *E. coli*. Furthermore, inflammation is required for such causal relationships between *E. coli* and microbiome, as no *E. coli* correlating genera were identified in the uninflamed intestine.

More importantly, we further revealed the identities of specific components underlying *E. coli*-mediated microbiome change, since compositional disruption can be linked to the intestinal dysfunction. For instance, in response to the expansion of *E. coli*, a subset protective lineage of Clostridiales (including *Ruminococcaceae* and *Lachnospiraceae*) were depleted, which has also been observed in IBD patients [14, 78]. As these lineages are involved in fermentation of complex dietary polysaccharides including fiber [80], we expect their disappearance will reduce the availability of short-chain fatty acids (SCFAs) necessary for maintenance of gut health [81, 82]. Unexpectedly, correlation revealed that *E. coli* promoted the expansion of probiotic *Lactobacillus*. It is possible that certain *Lactobacillus* might benefit from *E. coli*-derived metabolites [83]. While the above correlations were not shared with another cohort using FMT2 as an inoculum, *Enterococcus* always co-expanded with *E. coli* [11]. In concordance with their synergistic correlation pattern, dual association of *Il10*^*−/−*^ mice with *E. faecalis* and *E. coli* has been shown to trigger more aggressive intestinal inflammation than the degree induced by mono-association with each strain alone [32, 84]. In turn, inflammation can cause a perturbation in gut anaerobiosis, which facilitates expansion of these two facultative anaerobic lineages [85]. Taken together, these results support that *E. coli* expansion and intestinal inflammation both contribute to a dysbiotic mucosal microbiome and may drive compositional and even functional changes that enhance disease activity [86].

### The in vitro definition of AIEC may not predict mucosal colonization in vivo

Our ability to distinguish strain-level variations from among the complexity of the mucosally adherent microbiota allowed us to assess AIEC status in relation to colonization potential. Specific four strains persisting in WT and two strains persisting in *Il10*^*−/−*^ mice supports that the mucosal colonizers are not necessarily AIEC strains. There are likely non-AIEC strains that are more widely distributed than others in healthy and IBD-associated individuals [87, 88], and possibly as prevalent as CU39ES-1_A1 shown here. At a population level, although total numbers of non-AIEC strains dropped, CU39ES-1_A1 and CU37RT-2_D2 became remarkably more abundant as inflammation progressed in two respective *Il10*^*−/−*^ cohorts. In line with our observation in mouse models, certain non-AIEC strains can also persist at high levels in the mucosa of IBD patients [10, 14]. Indeed, our culture collection contains both AIEC and non-AIEC strains that were isolated from intestinal tissue of IBD patients. This observed dynamic and previous evidence imply that the expansion of non-AIEC colonizers can potentially contribute to inflammation, where non-AIEC cooperate with AIEC in *Il10*^*−/−*^ mice. Conversely, the “pathobiont” rather than “pathogenic” nature of these strains is evident as AIEC and non-AIEC strains failed to induce inflammation in non-susceptible WT mice [89]. The ability of non-AIEC to impair intestinal homeostasis could possibly be linked to putative virulence gene elements that are comparable to AIEC [28, 90, 91]. In this regard, mucosal colonization characteristics defined in vivo may better predict pro-inflammatory potential than the current in vitro AIEC definition, although additional future studies are needed. For example, it would be informative to evaluate adhesion and invasion to human intestinal organoids, as they would harbor the human-encoded adhesion factor CEACAM6 that binds *E. coli* FimH variants.

Taken together, our data suggest that the AIEC pathotype definition may not truly predict colonization phenotype, while high levels of non-AIEC strains can co-colonize with AIEC and contribute to dysbiosis and inflammation. Our work can be used as a guide to infer candidate colonization factors based on in vivo phenotype rather than AIEC classification.

### *E. coli* colonization patterns may be determined by external environmental factors and intrinsic genomic potential

Inflamed *Il10*^*−/−*^ mice harbored higher loads of colonizing *E. coli,* but with fewer strains retained than WT mice, indicating that inflammation may convey selective pressure for specific strains to colonize and expand. This is well linked to the previous observation that most AIEC-positive IBD patients carry two or less AIEC strains [92]. We found that two similar inoculum communities (FMT1 vs. FMT2) exhibited distinctive compositions at the final stage of colonization in the inflamed intestine. These FMT-associated microbiomes may have a bifurcating evolutionary trajectory that diversifies colonization dynamics of the co-evolved *E. coli* consortia. This is supported by different correlation patterns between microbiome and *E. coli* in the two related *Il10*^*−/−*^ cohorts colonized with either FMT1 or FMT2. These findings demonstrate the importance of external factors to determine the colonization fate of *E. coli* strains and necessitate future studies with additional animal models and diverse microbiomes.

Comparative genomics have been used to identify factors conferring metabolic fitness of AIEC strains; several responsible features were recently identified [93, 94]. Here we identify genetic determinants that may be important for mucosal colonization by *E. coli* in the inflamed intestine, relative to their colonization capacity and AIEC status. Although conducted on only seven strains, our pangenomic analysis revealed that the genomes of “colonizing” *E. coli* are enriched in genes for carbohydrate catabolism and iron acquisition relative to “disappearing” members. Western diet low in fiber confers an expansion of AIEC in the gut [95], and AIEC can reprogram its metabolic preference from carbohydrate to amino acids [96], thus “colonizing” *E. coli* strains may obtain a growth advantage over their commensal competitors by utilizing varied carbon metabolites through catabolism. Genes encoding iron acquisition via siderophores are overrepresented in AIEC vs. non-AIEC [93, 97], and iron is an important cofactor for metabolic enzymes that promote colonization of the gut. We observed dedicated iron-transporters were harbored by *E. coli* strains in the “colonized,” which may enhance their ability to outcompete neighboring microbes through iron acquisition. Remarkably, these potential colonization-promoting features were nearly all conserved between AIEC and non-AIEC strains, suggesting that a molecular definition of mucosal colonizers (rather than AIEC) should be further investigated. These studies will require mutating candidate colonization genes across multiple strains to determine their impact on colonization and inflammation.

Contrary to what we expected, we also observed that “colonizing” *E. coli* strains do not harbor T6SS and propanediol dehydratase that were harbored exclusively by the “disappearing” strains. This would suggest that T6SS and propanediol dehydratase are dispensable for *E. coli* to colonize the inflamed mucosa, but this conclusion differs from many published observations on *E. coli* persistence in the inflamed gut [93, 94, 97–99] and therefore warrants further study with a larger *E. coli* consortia. In fact, the distribution of T6SS is not restricted to specific pathotypes, and in other studies has been found in the genomes of multiple AIEC and non-AIEC strains [91, 94, 98]. Thus the impact of *E. coli* T6SS in colonization of the inflamed gut remains to be determined. Also in contrast to previous reports linking the ability to metabolize propanediol with virulence and persistence of AIEC in the gut [93, 97, 99], such crucial features were only specific to one “disappeared” strain. This discrepancy can potentially be explained by the defect in propanediol transformation being compensated with metabolism of other carbon sources. Also, it is important to point out that due to a lack of host cell adhesion molecule CEACAM6 on mouse epithelial cells [50], LF82_B6 failed to colonize the mucosa of inflamed *Il10*^*−/−*^ cohort, thus genotypic features of this strain may not be associated with the “disappeared” phenotype in a CEACAM6 + human host. Taken together, these initial pangenomic studies suggest that metabolic advantages harbored by in vivo colonizers do not necessarily correlate with those identified in the AIEC pathotype. We conclude that the *E. coli* intestinal colonization pattern is contingent on the combined effects of external co-evolved interactome and intrinsic metabolic flexibility.

## Conclusion

Mucosal colonization is essential for inflammation, a characteristic generally attributed to the in vitro defined AIEC pathovar. Yet it has been unclear whether in vitro AIEC definition can well explain colonization phenotype in vivo. Indeed, the lack of a genetic definition for AIEC could be due to a classification problem, where perhaps the in vitro definition must be refined by in vivo colonization studies. Our current study has developed and evaluated an innovative high-throughput in vivo approach that can overcome current technological limitations of distinguishing strain-level variations from among the complexity of the mucosally adherent microbiota. By using the mouse as a model for human intestinal *E. coli* colonization, we demonstrated the in vitro AIEC definition may not predict the mucosal colonizing dynamic of non-AIEC populations, while colonization phenotype is potentially driven by inherent metabolic plasticity under specific host contexts. Accordingly, our study highlights the centrality of *E. coli* mucosa colonizers, consisting of AIEC and non-AIEC, in dysbiosis-associated crosstalk between host and microbiome. Moreover, comparisons of genomes based upon colonization phenotype provide new insight into the genetic determinants of *E. coli* colonization of the mucosa, which we expect will improve the molecular identification of IBD patients harboring high-risk *E. coli,* who may be at risk for a complicated disease course.

### Supplementary Information

**Additional file 1.** **Supplementary Methods.**

**Additional file 2: Supplementary Figures S1-10.**

**Additional file 3: Supplementary Table S1.**

## Data Availability

The raw data for the 16S rRNA gene sequence and barcode sequence has been deposited in the NCBI BioProject database under accession number: PRJNA963181. The other data of this study are available on request from J.C.A.

## Abbreviations

AIEC: Adherent-invasive *Escherichia coli*
Anvi’o: Analysis and visualization platform for microbial ‘omics
CD: Crohn’s disease
CFU: Colony forming unit
COG: Clusters of Orthologous Groups
CRC: Colorectal cancer
*E. coli*: *Escherichia coli*
FMT: Fecal microbial transplant
IACUC: Institutional Animal Care and Use Committee
IBD: Inflammatory bowel disease
*Il10*^*−/−*^: Interleukin-10-deficient
KEGG: Kyoto Encyclopedia of Genes and Genomes
LB: Luria broth
MCL: Markov Clustering
PCoA: Principal Coordinate Analysis
SCFAs: Short-chain fatty acids
SEM: Standard error of mean
SPF: Specific pathogen free
T6SS: Type VI secretion system
UC: Ulcerative colitis
WT: Wild type
