# Supplementary material for: A consortia of clinical E. coli strains with distinct in vitro adherent/invasive properties establish their own co-colonization niche and shape the intestinal microbiota in inflammation-susceptible mice
Source: Microbiome. 2023 Dec 20;11:277. doi: 10.1186/s40168-023-01710-y (PMC10731797; doi:10.1186/s40168-023-01710-y)
Supplement: Supplementary file 2 — Additional file 1. Supplementary Methods. [file 40168_2023_1710_MOESM1_ESM.docx]

**Methods Supplemental Information**

***In vitro barcode sequencing (Fig S3)***

Murine AIEC strain NC101 was individually barcoded with 10 different molecular barcodes using Tn7 transposon insertion. The genome sequence of the NC101 parent strain is available at GenBank under accession number CP072787. To confirm that we could sequence and distinguish the barcodes, various known mixtures of these barcoded NC101 strains were pooled, from which DNA was extracted. These pools were sequenced and analyzed following the same protocol for the clinical isolates. Percent of total sequencing reads was calculated and compared to the expected ratios.

***In vivo transit experiments (Fig S3)***

To test there was no bottleneck in initial strain colonization, three male C57Bl/6 germ-free mice were gavaged with an even mixture of a total of 10^8^ CFU of five barcoded NC101 *E. coli* strains. Mice were sacrificed after 8 hours (1 mouse) or 24 hours (2 mice). Samples from fecal content were collected at the time of sacrifice and the relative abundance of each barcoded strain was determined by qPCR from stool DNA using barcode-specific primers as indicated below. Evenness of the barcoded strains was determined by calculating Pielou’s index.

***One week abundance (Fig S3)***

To verify strains initially colonize evenly, five barcoded strains of NC101 were evenly pooled and oral/rectal swabbed onto germ-free *Il10^-/-^* and wild-type 129S6/SvEv mice. Mice were maintained in sterile cages. One week after colonization with *E. coli*, stool samples were collected and were sequenced and analyzed following the same protocol for the clinical isolates.

***Presence/Absence PCR (Fig S4 and S10)***

Stool and tissue DNA were analyzed for presence/absence of the various barcoded strains. Individual PCR reactions targeting each barcode were performed using MyTaq polymerase following manufacturer’s instructions. Amplification was performed using a universal barcode forward primer (F1 in Fig. S1) and barcode-specific reverse primers for seven unique barcode sequences (A1/A3/B6/C2/C5/D2/D5 in Fig. S1). Data were collated as presence/absence of a visible band for each barcode within each mouse sample. Each time a PCR reaction was run, *E. coli* 16S rRNA primers were also used in parallel to validate the PCR cycling was successful.

***Quantification of barcoded strains by qPCR (Fig S4)***

Stool DNA (6 ng each) from 17 mice that received barcoded *E. coli* and FMT1 was subjected to qPCR with primer pairs targeting each barcode to quantify relative amounts of each isolate as previously described [1]. Amplification was performed in duplicate using SYBR green qPCR chemistry (Bioline) using the same primer sets in Presence/Absence PCR, with *E. coli* 16S primers (F-5'-ATTGACGTTACCCGCAGAAGA-3', R-5'-GGGATTTCACATCCGACTTGA-3') [2, 3] on a QuantStudio 6 Real-Time PCR System. Ct values were normalized to *E. coli* 16S rRNA to generate ΔCt values, and fold changes were calculated by ΔΔCt to the ΔCt of the mouse inoculum.

***Growth curves (Fig S4)***

Growth curves were performed to ensure that barcode insertion did not impact bacterial growth, as expected. Clinical *E. coli* strains with or without barcode inserted were grown in M9 minimal media as previously described [4]. OD_600_ measurements were taken every 60-90 minutes over the course of 7 hours.

***Impact of 2-week oral kanamycin on microbiome (Fig S5)***

Our goal was to minimally disrupt the microbiota, but allow barcoded kanamycin-resistant *E. coli* isolates to maintain colonization in the presence of a murine microbiota. Accordingly, to test the impact of kanamycin on the microbiota, *Il10^-/-^* mice were reared germ-free to adulthood (8-10 weeks) in cohorts that received kanamycin (0.4 g/L; JA134) and did not receive kanamycin (JA156) listed in Figure 1. Mice were maintained in SPF housing, and given 100 µL of thawed FMT for colonization. Two weeks post-FMT, we gave kanamycin water *ad libetum* to one cohort for 2 weeks while the other stayed on regular water. Mice were harvested 6 weeks later by CO_2_ asphyxiation for a total of 10 weeks colonization post-FMT. Stool samples were removed from the lumen and 1 cm of colonic mucosal samples were taken for sequencing analysis. Sequencing and histological analysis was performed as described in the main text.

***Quantification of cytokine expression by qPCR (Fig S6)***

Pro-inflammatory cytokine transcript levels in colon tissue represent a molecular marker of inflammation. One centimeter of proximal colon tissue from 13 mice that received FMT1 only, and 7 mice that received barcoded *E. coli* and FMT1, was transferred into 1 ml Trizol (Invitrogen) and RNA was extracted following the manufacturer’s protocol. Isolated RNA was subjected to DnaseI treatment (Invitrogen) prior to cDNA synthesis. cDNA synthesis was completed using qScript cDNA SuperMix (Quantabio). qPCR amplification was performed in duplicate with SYBR green qPCR chemistry (Bioline) using primers for *Tnfa* (F-5'-ACCTCACACTCAGATCATCTTCTC-3', R-5'-TGAGATCCATGCCGTTGG-3'), *Il1b* (F-5'-ACAGAATATCAACCAACAAGTGATATTCTC-3', R-5'-GATTCTTTCCTTTGAGGCCCA-3'), *Il12B(p40)* (F-5'-CGCAAGAAAGAAAAGATGAAGGAG-3', R-5'-TTGCATTGGACTTCGGTAGATG-3'), *Il6* (F-5'-GAAATGATGGATGCTACCAAACTG-3', R-5'-CTCTCTGAAGGACTCTGGCTTG-3'), *Il17a* (F-5'-AACCGTTCCACGTCACCCTGGA-3', R-5'-TGGTCCAGCTTTCCCTCCGCA-3'), and *Gapdh* (F-5'-GGTGAAGGTCGGAGTCAACGGA-3', R-5'-GAGGGATCTCGCTCCTGGAAGA-3') on a QuantStudio 6 Real-Time PCR System. *C_t_* values were normalized to *Gapdh* to generate Δ*C_t_* values, and fold changes were calculated by ΔΔ*C_t_* to the Δ*C_t_* of the WT mice that received barcoded *E. coli* and FMT1.

***Comparisons of microbial taxonomic abundance between FMT1 and FMT2 (Fig S7)***

FMT2 was prepared separately from the initial FMT1 as described in main method. Two groups of cohorts of germ-free mice were respectively treated with FMT1 or FMT2 (four cohorts per group separated by dashline in Figure 1). To facilitate unbiased comparisons of the fecal and mucosal microbiota between these two groups of cohorts, the similarity of their inocula (FMT1 vs. FMT2) were assessed in compositional barplot and correlation dotplot at the genus level. Genera counts were normalized and log_10_ transformed according to the same formula as previously described [5].

***Correlation between Enterococcus and Escherichia abundance in different cohorts (Fig S9)***

Spearman’s rank-order correlations showed that the abundance of *Enterococcus* was positively correlated with *Escherichia* in certain cohorts, which were supported by FDR-corrected *p*-values (Figure 6). To display such relationship in an intuitional manner, the log normalized counts of both genera from each of five cohorts were plotted, and resolved into fecal and mucosal niches.

**References**

1. Wang J, Bleich RM, Zarmer S, Zhang S, Dogan B, Simpson KW, et al. Long-read sequencing to interrogate strain-level variation among adherent-invasive Escherichia coli isolated from human intestinal tissue. PLoS One. 2021;16(10):e0259141; doi: 10.1371/journal.pone.0259141.

2. Gonzalez RJ, Lane MC, Wagner NJ, Weening EH, Miller VL. Dissemination of a highly virulent pathogen: tracking the early events that define infection. PLoS Pathog. 2015;11(1):e1004587; doi: 10.1371/journal.ppat.1004587.

3. Walters MS, Lane MC, Vigil PD, Smith SN, Walk ST, Mobley HL. Kinetics of uropathogenic Escherichia coli metapopulation movement during urinary tract infection. mBio. 2012;3(1); doi: 10.1128/mBio.00303-11.

4. Lopez LR, Barlogio CJ, Broberg CA, Wang J, Arthur JC. A nadA mutation confers nicotinic acid auxotrophy in pro-carcinogenic intestinal Escherichia coli NC101. Front Microbiol. 2021;12:670005; doi: 10.3389/fmicb.2021.670005.

5. Ellermann M, Gharaibeh RZ, Maharshak N, Perez-Chanona E, Jobin C, Carroll IM, et al. Dietary iron variably modulates assembly of the intestinal microbiota in colitis-resistant and colitis-susceptible mice. Gut Microbes. 2020;11(1):32-50; doi: 10.1080/19490976.2019.1599794.
