# Supplementary material for: A consortia of clinical E. coli strains with distinct in vitro adherent/invasive properties establish their own co-colonization niche and shape the intestinal microbiota in inflammation-susceptible mice
Source: Microbiome. 2023 Dec 20;11:277. doi: 10.1186/s40168-023-01710-y (PMC10731797; doi:10.1186/s40168-023-01710-y)
Supplement: Supplementary file 3 — Additional file 2: Supplementary Figures S1-10. [file 40168_2023_1710_MOESM2_ESM.docx]

**Fig S1. Barcoding approach to examine *in vivo* colonization potential of individual *E. coli* isolates. A.** Transformation of *E. coli* strain with Tn7 transposon to insert barcode sequence (~ 60 bp) into its chromosome. Sequences of the 10 unique barcodes used in this study, with region for barcode-specific primer binding highlighted in red. Sequencing primers (F1/P2/P4) and barcode-specific primers in the table were used for verification of successful insertion. **B.** Invasion of cultured Caco-2 intestinal epithelial cells by barcoded *E. coli* strains and parental controls. **C.** Persistence and replication in J774 macrophages. Values 100% are consistent with replication. The results shown are Mean ± SD of triplicates from two independent experiments.

**Fig S2. Inflammatory cytokine expression in the proximal colon of *Il10^-/-^* mice as assessed by qRT-PCR.** **A-C**. Duplicate PCoA plots (of Figure 2C-D) and average colitis histology score (of Figure 3B but where mouse 17 was not displayed) marked with both mouse ID and time of sacrifice. **D.** Relative mRNA abundance of each cytokine from the *Il10^-/-^* mice that received with FMT1 and 7 *E. coli* isolates that were sacrificed early (blue, n = 4) or survived the full 10 weeks (orange, n = 4). Note: There is a missing data point for *Il1b* early, *Il12b* early and late, as a result of a PCR failure on low amount of expression. All values were normalized to *gapdh* and fold change was calculated relatively to the WT mice that received with FMT1 and 7 *E. coli* isolates. Line represents the median and *p*-values determined by Mann-Whitney test (* *p* < 0.05). This is provided to demonstrate that while the mice necessitated sacrifice early, their colitis was still in range with the other animals (based on histology score and cytokine expression), thus they were included in the dissimilarity analysis.

**Fig S3. *In vitro* (A) and *In vivo* (B-E) verification of barcode sequencing using NC101 *E. coli* barcoded strains. A.** Actual and expected abundance of barcoded sequences based on sequencing five mixtures of known ratios of ten differently barcoded NC101strains. **B.** Estimated timeline showing when *E. coli* reach different regions of GI tract in a 24 hour *in vivo* colonization experiment. **C.** Percent abundance of barcoded sequences based on qPCR of stools in germ-free mice at sacrifice 8 (n=1) or 24 (n=2) hours post-gavage with even mixture of five differently barcoded NC101 strains. **D.** Evenness was determined by Pielou’s index from the same dataset in C. **E.** Abundance of barcoded sequences based on sequencing stools in germ-free mice (n=13) at one-week post-colonization (via oral/rectal swab) with NC101 strains. * denotes five WT mice, and the remaining are *Il10^-/-^* mice.

**Fig S4. *In vivo* comparison of three barcode detection methods (A-C) on the same stool samples of *Il10^-/-^* mice that received 7 *E. coli* isolates (described in Figure 1) and FMT1 (cohort JA123: *Il10^-/-^*+7*E.coli*+FMT1). A. Presence/absence analysis:** The number of mice where the presence of inserted barcodes (indicated by black bars) was detected by PCR using a standard protocol with the primers listed in Fig S1. Samples in A-C are the same DNA subjected to Illumina sequencing, collected at harvest. **B.** Fold change of barcoded reads relative to the even inoculation pool determined by qPCR. **C.** Abundance (normalized and log-transformed) of barcoded sequences obtained by Illumina sequencing. Three barcode sequences (A4/B2/D1) that had not been inserted into *E. coli* were queried, but not detected by any of the three methods. **D.** Growth curves of 7 *E. coli* strains with or without barcodes in M9 minimal media.

**
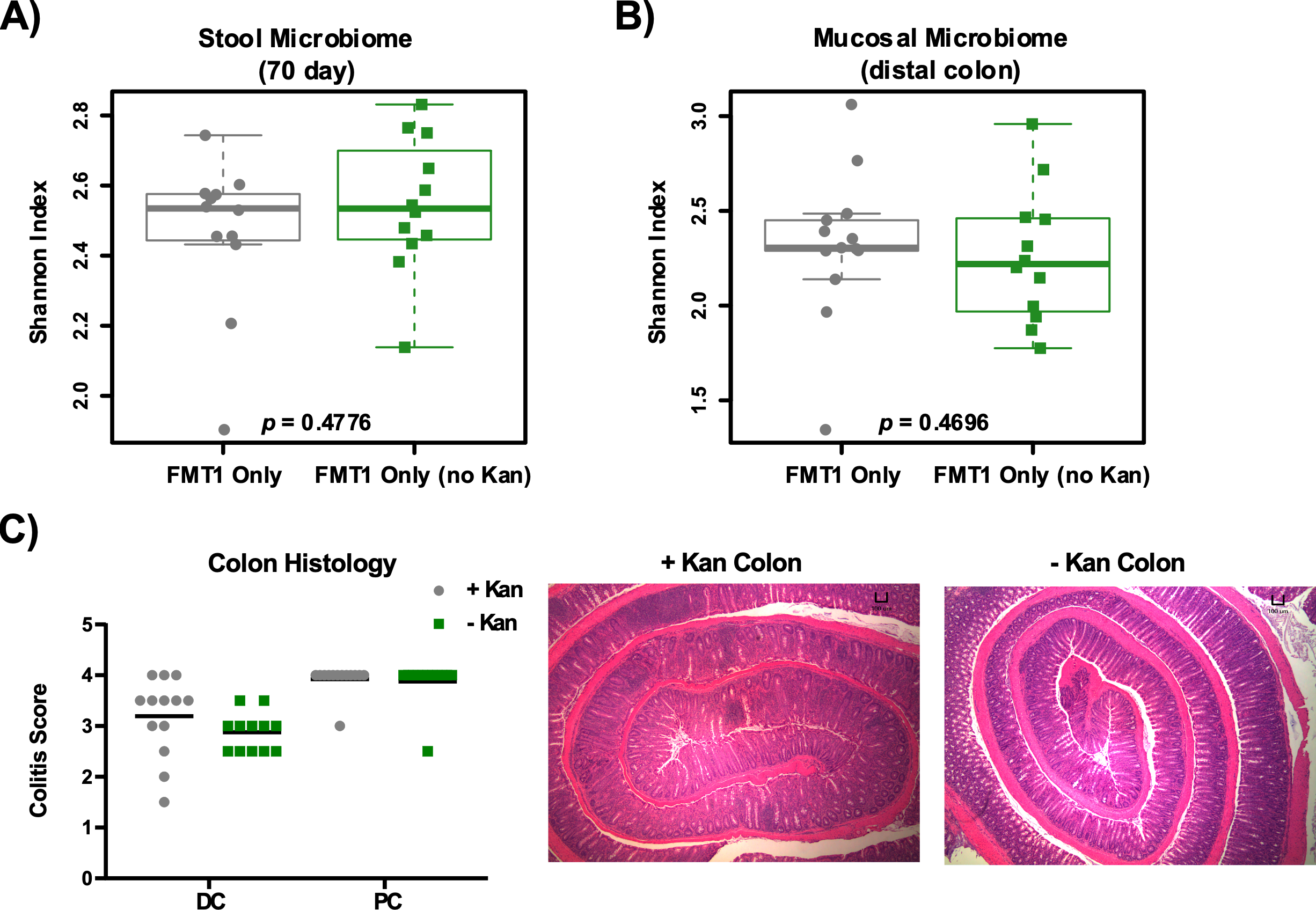
**

**Fig S5. Kanamycin (Kan) administration had minimal impact on the stool and mucosal microbiota.** **A and B.** Shannon diversity index for the stool (A) and distal colon mucosal (B) microbiota in *Il10^-/-^* mice that received FMT1 with or without Kan from weeks 2-4 post-gavage (see timeline in Figure 1). Each symbol represents an individual mouse, with no significant difference between cohorts. **C.** Inflammation of the distal colon (DC) and proximal colon (PC) in *Il10^-/-^* mice was assessed by histologic scores (0-4) and compared with the score in mice with or without Kan. Line is at mean, and no significant difference between treatments. Representative H&E histology at 40X of the colon. Scale bar = 100 µm.

**Fig S6. Inflammatory cytokine expression in the proximal colon of *Il10^-/-^* mice as assessed by qRT-PCR.** Relative mRNA abundance of each cytokine from the *Il10^-/-^* mice that received with FMT1 and with (red) or without (blue) 7 *E. coli* isolates. Note: All values were normalized to *gapdh* and fold change was calculated relatively to the WT mice that received with FMT1 and 7 *E. coli* isolates. Line represents the median and *p*-values determined by Mann-Whitney test (* *p* < 0.05).

**Fig S7. Similar composition of microbial communities in the initial FMT1 and FMT2 inoculum.** **A.** Barplot showing relative abundance of DNA sequences from different bacterial genera present in the FMT1 and FMT2 inocula. Genera with < 1% abundance in either of the two FMTs were collapsed together, and sorted according to the abundance in FMT1. **B.** Dotplot showing absolute abundance (normalized and log-transformed) of specific bacterial genera (one genus for each dot) contained in the different FMT inoculum.

**Fig S8. PCoA based on Bray-Curtis distance for the mucosal (A-C) and stool (D-F) microbiota in *Il10^-/-^* mice that received with 3 vs 7 (A&D), 3 vs 1 (B&E), or 7 vs 1 (C&F) *E. coli* isolate(s) along with FMT2.** Non-significant *p*-values by PERMANOVA indicate the number of unique *E coli* strains initially inoculated into the mouse is not the key factor for modulating the structure of the accompanied intestinal microbiota.

**Fig S9. Correlation between *Enterococcus* and *Escherichia* abundance in (A and B) WT vs *Il10^-/-^* mice that received with 7 *E. coli* isolates and with FMT1 (C and D) *Il10^-/-^* mice with 7 vs 3 vs 1 *E. coli* isolate(s) and with FMT2. A and B.** Plot of abundance (normalized and log-transformed) of *Enterococcus* and *Escherichia* in the mucosa (A) and stool (B) from *Il10^-/-^* (blue) and WT (purple) mice reveal potential correlation in *Il10^-/-^* but not WT mice**. C and D**. Plot of abundance (normalized and log-transformed) of *Enterococcus* and *Escherichia* in the mucosa (C) and stool (D) from mice innoculated with 7 (blue) vs 3 (red) vs 1 (green) *E. coli* isolate(s).

**Fig S10. Barcode detection in GI tissues of *Il10^-/-^* mice that received 7 *E. coli* isolates and FMT1 (mice model: *Il10^-/-^*+7*E.coli*+FMT1).** Bar plots showing the number of mice where the presence of inserted barcodes was detected by PCR for the distal colon mucosa (A) and ileal tissue (B). * denotes the AIEC strains.
